# OLDER WOMEN ENTREPRENEURS ARE DRIVEN BY WANT RATHER THAN NEED

**DOI:** 10.1093/geroni/igad104.1920

**Published:** 2023-12-21

**Authors:** Lona Choi-Allum

**Affiliations:** AARP, Washington, District of Columbia, United States

## Abstract

During the COVID-19 pandemic, women ages 50+ pursued their dream of starting a business. This study contacted 278 women ages 50-plus by phone and online, examining the drivers and barriers of women entrepreneurs. Over a quarter (27%) of women said they always wanted to start a business, and 19% said they did it to follow their passion; another 17% were pursuing additional income, and 11% wanted flexible work options. Age, and perhaps the value of experience, has been an advantage in business ownership. Women entrepreneurs age 50-plus were less likely to have faced financial challenges since starting their business, with over two in five (45%) avoiding such challenges, compared to 29% of women entrepreneurs in their 40s. Nearly seven in 10 women (69%) surveyed poured their personal savings into their start-up. In addition, two in three agree that they face unique challenges in trying to access capital for their business that are different from men. Despite these challenges, most women were optimistic about their entrepreneurial path. The majority of women (97%) agreed that they made the right decision in starting their business – with about two in five (39%) saying their business is doing better than expected compared to when they first started. Respondents say they need resources on marketing, recruiting and hiring staff, and financing. And over two in five say they have not taken any type of training. Increasing awareness of business supports, funding sources, and training opportunities will help women as they grow their business.

